# Un “boxing” the reality: Knowledge, attitude, and perception of orthodontists, dental practitioners, and orthodontic patients toward mouthguards' wear during contact sports—A cross‐sectional study

**DOI:** 10.1002/cre2.904

**Published:** 2024-06-04

**Authors:** Shailaja Raghavan, Elham S. Abu Alhaija, Yousef Nasrawi, Susan Al‐ Khateeb, Samer Sunna

**Affiliations:** ^1^ College of Dental Medicine, QU Health Qatar University Doha Qatar; ^2^ Orthodontics and Dentofacial Orthopedics Department, Henry M. Goldman School of Dental Medicine Boston University Massachusetts Boston United States; ^3^ Department of Preventive Dentistry, Division of Orthodontics, School of Dentistry Jordan University of Science and Technology Irbid Jordan; ^4^ Sunna Orthodontics Amman Jordan

**Keywords:** contact sports, mouthguards, orthodontic treatment

## Abstract

**Introduction:**

Mouthguards (MGs) have the potential to prevent contact sport‐related dental injuries. However, varying perceptions of their effectiveness persist, influencing recommendations by dental professionals.

**Aim:**

To assess the attitudes, knowledge, and perceptions of orthodontists, other dental practitioners (general dentists and other dental specialists), and orthodontic patients involved in contact sports regarding the use of MGs.

**Methodology:**

A cross‐sectional survey was designed to collect information from dental clinicians (orthodontists and other dental practitioners) and their orthodontic patients about using MGs during sports participation. A convenience sampling technique was used to recruit the participants for an online survey. A total of 107 (32 males/75 females) dental clinicians and 147 (75 males/72 females) orthodontic patients (mean age 17.5 ± 5.84 years) participated in the study. Pre‐validated questionnaires, specifically designed for dental clinicians and orthodontic patients, were used. Data were analyzed using SPSS software (version 28.0; SPSS).

**Results:**

Nearly 50% of dental clinicians have recently recommended MGs to their patients in their clinical practice out of which 59% preferred the stock type; 33% of dental clinicians enquired their patients about involvement in contact sports during initial visits. The majority of orthodontic patients acquired knowledge about MGs through the Internet. More than half of orthodontic patients expressed unwillingness to pay for MGs, and 89% of orthodontic patients found using MGs during contact sports uncomfortable.

**Conclusion:**

The findings provide valuable insights into the practices and attitudes of dental clinicians and orthodontic patients regarding MGs, their recommendations, and the comfort levels associated with using them during contact sports.

## 1 INTRODUCTION

With an increase in the quantum of involvement in sports activities, especially among children and adolescents, there has been a proportional increase in the risk of trauma to the orofacial structures. The prevalence of orofacial and dental trauma has been reported to range between 20% and 60% and is attributed to the orientation of anatomic structures that makes them vulnerable to the impact/trauma during sports activities (Bruggesser et al., 2020; Petrović et al., 2016). Complimenting this fact, the combined prevalence of orofacial and dental sports‐related injuries was determined to be 40.6% (Tewari et al., 2023)

The majority of orthodontic treatment occurs either before or during adolescence, a period marked by an increase in the occurrence of dental injuries during participation in contact sports (Love et al., 1998). Orthodontic patients who participate in contact sports during their course of fixed appliance therapy may be more liable to orofacial injuries as a result of loosening or debonding of brackets, arch wire distortion, damage to the dento‐alveolar structures, and soft tissue laceration (Maeda et al., 2008). Furthermore, a recent study has emphasized the significance of the presence and type of orthodontic brackets in influencing the stress and strain experienced during traumatic impacts (Alves et al., 2020).

Nevertheless, it is believed that some of the contact sport‐related dental trauma can be prevented by wearing a mouthguard (MG) as it sustains the impact of trauma and dissipates that energy, which would otherwise be detrimental to the underlying dentition (Newsome et al., 2001; Owtad et al., 2015). There are three types of MGs, namely pre‐fabricated MGs (stock MGs, which are not customized for the patient), mouth‐formed or “boil‐and‐bite” MGs (crafted from a thermoplastic material that becomes pliable when heated and shaped by the wearer), and custom‐made MGs (fabricated using dental impressions).

The American Dental Association (ADA) has established guidelines specifying the particular sports that necessitate the use of MGs (Using, 2006). Moreover, a meta‐analysis suggests that the likelihood of experiencing an orofacial injury is approximately two times greater when not using an MG during sport (Knapik et al., 2007). Despite the protective nature of MGs, there is still a reluctance to wear them during sports activities, even among players who are aware of their benefits (Bergman et al., 2017; O'Malley et al., 2012; Tiryaki et al., 2017). This reluctance may be attributed to challenges associated with carrying out normal physiological functions, among other factors.

Notably, persistent divergence in the overall perception of the effectiveness and utility of MGs exists, which affects recommendations by dental professionals (Bastian et al., 2020; Bussell & Barreto, 2014). Various factors contribute to this divergence, including their beliefs, the type of sport, patients' requests, treatment acceptance, patient disclosure of participation in contact sports, the nature of malocclusion, the level and frequency of sports involvement, the influence of previous dentist, and their clinical exposure to patients presented with traumatic injuries (Bastian et al., 2020; Bussell & Barreto, 2014). While several studies have explored players' views, (Chapman, 1990; Galic et al., 2018; Hayashi et al., 2020; Lieger & von Arx, 2006; Meyfarth et al., 2023; O'Malley et al., 2012; Sarao et al., 2021) data are scarce regarding orthodontists' and other dental professionals' standpoint regarding the importance of MGs and their use by athlete patients undergoing orthodontic treatment.

Hence, the aim of this cross‐sectional study was:

– To report on the attitude, knowledge, and perception of orthodontists and other dental practitioners (general dentists and other dental specialists) toward MGs.
– To report on the attitude and perception of orthodontic patients involved in contact‐sport activities toward the use of MGs.

## 2 MATERIALS AND METHOD

### 2.1 Study design

This cross‐sectional survey was designed to determine the knowledge, perceptions, practice, and attitudes of dental clinicians (orthodontists and other dental practitioners) and their orthodontic patients toward the use of mouth guards during sports participation. The protocol was approved by the Deanship of Research/Jordan University of Science and Technology (Research ID20070048).

### 2.2 Eligibility criteria

The eligibility requirements encompassed the following conditions: dental clinicians providing orthodontic treatment to patients and patients undergoing orthodontic treatment, possessing the capability to access online content in English, and providing informed consent for the utilization of the recorded data.

### 2.3 Recruitment

A convenience sampling technique was used to recruit participants for the study. Self‐administered Google Forms questionnaires were circulated between January and March 2023. The surveys were circulated through emails and WhatsApp groups to maximize the response rate and to include a diverse population. The participants included were orthodontists, their orthodontic patients, and dental practitioners (DP) in Jordan. Out of 152 dental specialists who received the survey link, 124 responded and once the data had been cleaned 107 responses were considered for the final analysis (response rate 81.6%).

A total of 107 (32 males/75 females) dental clinicians with varying years of experience from various clinical settings (governmental and private practices) participated in an online survey.

The survey included orthodontic patients who had received treatment from the participating dental clinicians. Out of 174 patients who received the survey link, 157 responded, and once the data had been cleaned 147 responses were considered for the final analysis (response rate 90%).

One hundred forty‐seven orthodontic patients (75 Males/72 Females) within the age range of 14–20 (mean age 17.5 ± 5.84 years) who were undergoing active orthodontic treatment (either with removable or fixed orthodontic appliances) and involved in active sports participated in the study.

### 2.4 Instrumentation

A pre‐validated 13‐item and a 10‐item questionnaire, (Bastian et al., 2020; Bussell & Barreto, 2014) tailored specifically for dental clinicians and orthodontic patients respectively were circulated through e‐mails and WhatsApp messages. Some of the questions, which were extracted from the previously conducted qualitative study (Bussell & Barreto, 2014; Lieger & von Arx, 2006), underwent reformulation by the researchers and were included in the questionnaire. All participants provided informed consent as part of the questionnaire. The questionnaire included multiple‐choice questions (MCQs).

### 2.5 Data collection

Data was collected through an online questionnaire disseminated to participants. The purpose of the survey was explained to the participants; it was emphasized, to the patients, that survey participation was voluntary, their participation would not affect their treatment and at any time they could stop taking the survey. Participation in the survey was anonymous and responses were kept confidential.

### 2.6 Statistical analysis

Statistical analysis was performed using SPSS software (version 28.0; SPSS). Descriptive statistics were computed for demographic information as well as individual patient responses. Data were analyzed statistically using the Chi‐square test with a 95% confidence interval. *p* < 0.05 was considered statistically significant.

## 3 RESULTS

### 3.1 Dental clinicians

Out of the 107 dental professionals who completed the survey, 48.6% were orthodontists, 33.6% were general dental practitioners (who work in an orthodontic practice and provide orthodontic treatment with removable appliances), and 19% were dental specialists other than orthodontists (endodontists, prosthodontists, pediatric dentists). Of those, 38% had more than 10 years, 30% had more than 5 years, and 32% had less than 5 years of professional experience.

#### 3.1.1 Current clinical practice and attitude

The responses from the participants are shown in Table 1.

**Table 1:** Responses of dental practitioners and Orthodontists to each item based on current clinical practice, attitude, perception, and knowledge about mouthguards.

Items	Responses	Frequency distribution counts (%)		P value
		DP	Orthodontists	
**CURRENT CLINICAL PRACTICE AND ATTITUDE**				
1. In your dental practice, do you routinely ask your patients whether they play sports/contact sports as a part of history during the initial visit?	Yes	20 (36.4)	15 (28.8)	0.407
	No	35 (63.6)	37 (71.2)	
2. Have you recommended a mouthguard to any of your patients in practice recently?	Yes	27 (49.1)	22 (42.3)	0.482
	No	28 (50.9)	30 (57.7)	
3. If recommended in your clinical practice, which is the one advised routinely?	Stock type	19 (70)	10 (45)	0.067
	Boil and bite type	0	3 (14)	
	Custom made	8 (30)	9 (41)	
4. Have your patients reported any discomfort while using the mouthguard?	Yes	20 (74)	8 (36)	0.007*
	No	7 (26)	14 (64)	
5. Have any of your patients reported trauma to the teeth while using mouthguards?	Yes	5 (19)	2 (9)	0.348
	No	22 (81)	20 (91)	
6. Do you have an in‐office facility to fabricate custom‐made mouthguards?	Yes	14 (25)	17 (33)	0.409
	No	41 (75)	35 (67)	
7. Do you charge your patients for the custom‐made mouthguards?	Yes	13 (93)	13 (76)	0.217
	No	1 (7)	4 (24)	
8. If you found or/patient informed you that he/she is playing contact sport, would you recommend him/her to use of mouthguard during contact sport activities?	Yes	47 (85)	41 (79)	0.371
	No	8 (15)	11 (21)	
9. Do you think it is necessary to get the patient's consent before recommending it?	Yes	40 (73)	29 (56)	0.067
	No	15 (27)	23 (44)	
10. For orthodontic patients who play contact sports, whom do you think should make decisions regarding the use of mouthguards?	Orthodontist	46 (83.6)	38 (73.1)	0.120
	General dentist	5 (9.1)	2 (3.8)	
	Coach	4 (7.3)	9 (17.3)	
	Parents	0 (0)	2 (3.8)	
**PERCEPTION AND KNOWLEDGE ABOUT MOUTHGUARDS DURING ORTHODONTIC TREATMENT**				
11. What would be your specific recommendation regarding the use of mouthguards in patients with removable appliances/functional appliances?	Remove the appliance and wear a mouthguard during sport	50 (91)	43 (83)	0.125
	Refrain from the sport until treatment completion	4 (7)	3 (5)	
	Mouthguard is not necessary during active sporting	1 (2)	6 (12)	
12. What would be your specific recommendation regarding the use of mouthguards in patients with fixed appliances?	Wear a mouthguard during sport	48 (87)	43 (83)	0.092
	Refrain from the sport until treatment completion	6 (11)	3 (5)	
	Mouthguard is not necessary during active sporting	1 (2)	6 (12)	
13. Which sport do you think necessitates the use of a mouthguard?	Foot ball	1 (1.8)	1 (1.9)	0.472
	Basket ball	1 (1.8)	1 (1.9)	
	Hockey	2 (3.6)	1 (1.9)	
	Volley ball	2 (3.6)	1 (1.9)	
	All contact sports	48 (87.3)	42 (90.4)	
	Not necessary at all	1 (1.8)	6 (1.9)	

* Statistically significant

Out of 107 respondents, almost one‐half (46%) of dental clinicians (55% DP and 45% Orthodontists) recommended MGs to their patients in their practice recently; 38% of the clinicians had more than 10 years, 30% had more than 5 years, and 32% had less than 5 years of professional experience. Among all the respondents, 33% of dental clinicians (57% DP and 43% Orthodontists) reported that they routinely enquire about their patients' engagement in contact sports as part of history assessment during the initial consultation. When questioned whether they would recommend the use of MG if they were informed by their patients later during treatment about their involvement in contact sports, 82% of them (53% DP and 47% Ortho) expressed a positive response. Almost two‐thirds of the participants (64%) believed that obtaining patient consent is essential before making a recommendation for the use of MGs.

**Figure 1:**
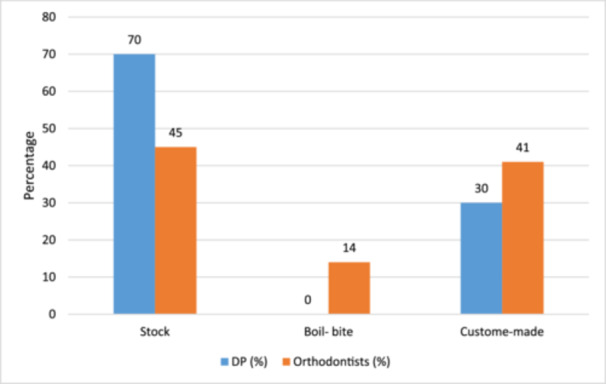
Type of mouthguards recommended by dental clinicians in their practice recently.

Of those who recently recommended MGs for their patients, 59% (66% DP and 34% Orthodontist) recommended the use of stock‐type MGs (Figure 1). Among all the respondents, only 29% (45% DP and 55% Orthodontists) indicated that they have in‐office MG fabrication facilities and of those, 84% mentioned that they would charge the patient for this service.

Of the dental clinicians who routinely recommended the use of MG during contact sports, 57% of them (71% DP and 29% Orthodontists) reported that their patients had complained of discomfort while wearing the MGs and the difference between DP and Orthodontists was statistically significant (*p* = 0.007). Additionally, 14% of them stated that their patients experienced trauma despite using the MG.

#### 3.1.2 Dental clinicians' perception and knowledge about MG during orthodontic treatment

The majority of dental clinicians (84%) believed that the use of MGs is necessary and to be worn by orthodontic patients involved in all forms of contact sports. However, a few dental clinicians prioritize certain contact sports over others when it comes to mandating the use of MGs.

**Figure 2:**
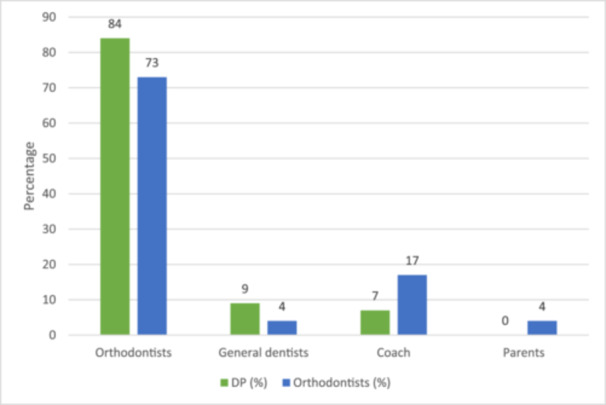
Perceptions of dental professionals concerning their role in decision‐making for recommending mouthguards to orthodontic patients.

Of all the dental practitioners, 79% (55% DP and 45% Orthodontists) felt that the decision‐making with regard to the recommendation of MGs to orthodontic patients who actively play contact sports should primarily rest on orthodontists (Figure 2).

**Figure 3:**
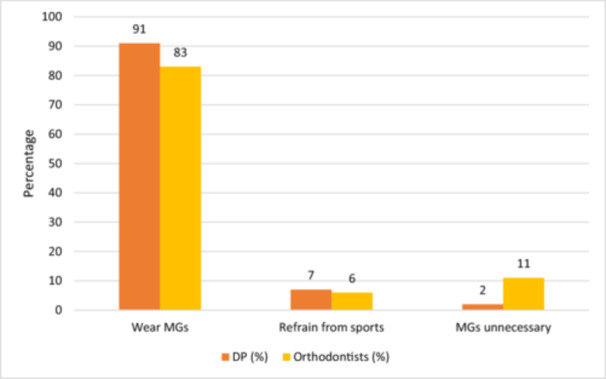
Dental Clinicians' perspectives on recommending mouthguards (MGs) for patients with removable appliances.

**Figure 4:**
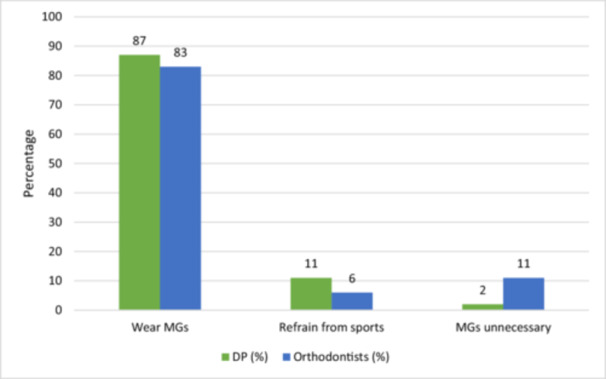
Dental clinicians' perspectives on recommending mouthguards (MGs) for patients with fixed appliance.

Based on the orthodontic appliance type, the majority of dental practitioners claimed that they would advise their orthodontic patients with removable (87%) and fixed (85%) appliances to wear MGs during contact sports while the others recommended otherwise (Figures 3 and 4).

There were no statistically significant differences in most of the responses between the orthodontists and DP.

### 3.2 Patients

**Figure 5:**
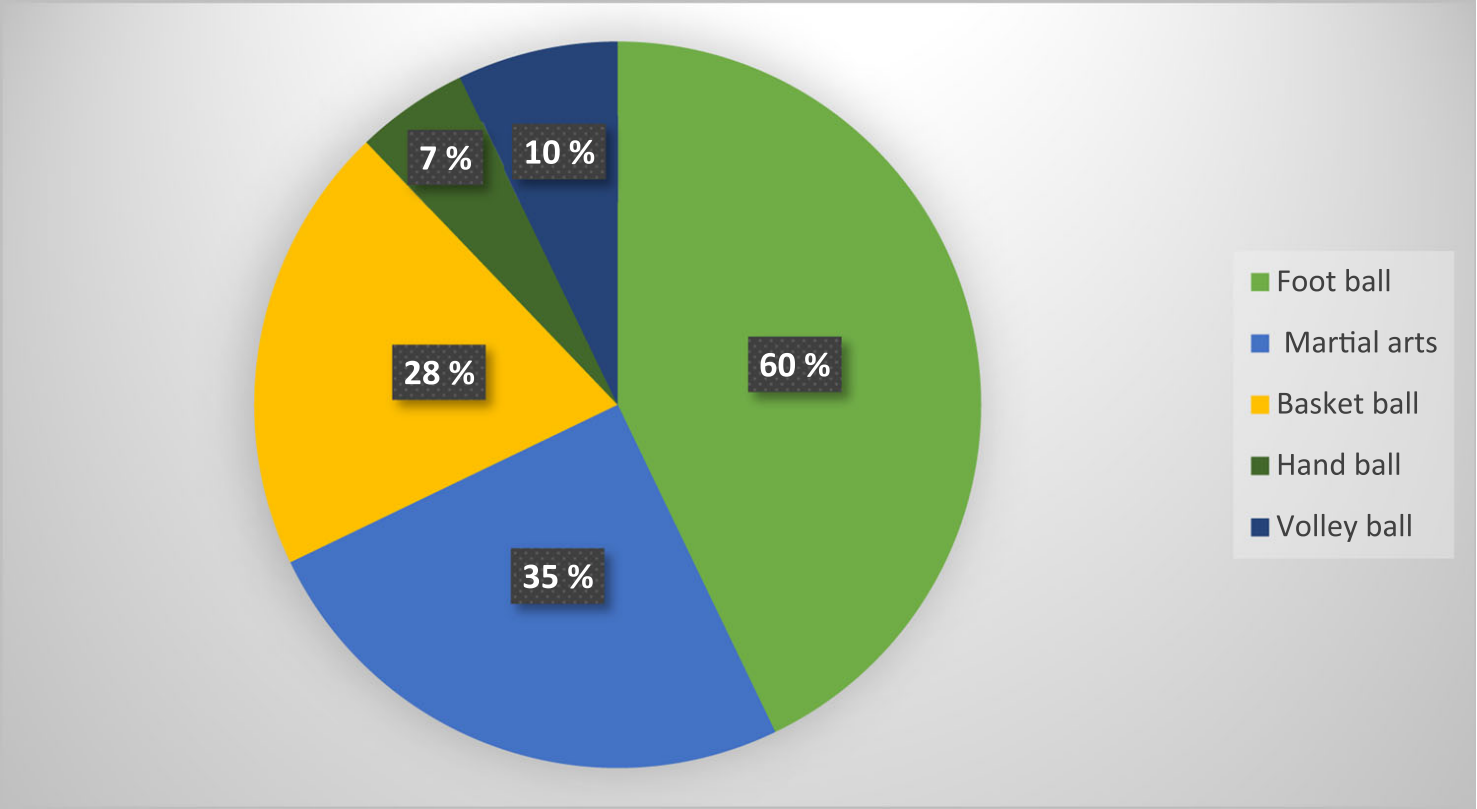
Distribution of orthodontic patients by the type of contact sports involved.

The descriptive data and the responses from the participants are shown in Tables 2 and 3 respectively. The distribution of subjects based on the type of sport is shown in Figure 5.

**Table 2:** Demographic characteristics of the study population (orthodontic patients involved in contact sports).

Age	17.56 (5.84) years	
Gender	Females	72 (49%)
	Males	75 (51%)
Contact sports involved	Football	60 (40.8%)
	Martial arts	35 (23.8%)
	Basketball	28 (19%)
	Volley ball	10 (6.8%)
	Hand ball	7 (4.8%)
Type of appliance	Fixed	113 (76.9%)
	Removable	34 (23.1)
Duration of appliance wear	Less than 6 months	35 (23.8%)
	6months to year	62 (42.2%)
	More than one year	50 (34%)

**Table 3:** Responses of orthodontic patients (involved in contact sports) to each item based on attitude and perception about mouthguards.

Items	Response	Frequency distribution counts (%)	Total responses
1. Have you experienced any trauma to your teeth or mouth during contact sports before	Yes	65 (44.2%)	127
	No	62 (55.8%)	
3. Did you hear about mouthguards to protect your teeth and mouth during contact sports before	Yes	107 (72.8%)	147
	No	40 (27.2)	
5. Have you seen any mouth guards before	Yes	83 (56.5%)	147
	No	64 (43.5%)	
7. If yes to the above question, where did you see it	Orthodontist	10 (6.8%)	94
	Coach	24 (16.3%)	
	General dentist	9 (6.1%)	
	Friends	21 (14.3%)	
	Internet and social media	30 (20.4%)	
12. Do you wear a mouthguard during contact sports routinely	Yes	57 (38.8%)	147
	No	90 (60.2%)	
14. Will you be cooperative with wearing a mouthguard during contact sports if it is recommended for you?	Yes	120 (81.6%)	147
	No	27 (18.4%)	
16. Are you prepared to pay additional costs for the mouthguard to protect your teeth during contact sports?	Yes	71 (48.3%)	147
	No	76 (52.7%)	
17. Do you believe that a mouthguard will protect your teeth during contact sports?	Yes	127 (86.4%)	147
	No	20 (13.6%)	
19. Do you think wearing the mouthguard during sports is uncomfortable?	Yes	112 (89.8%)	120
	No	15 (10.2%)	
21. In general, why do you think the mouthguard is not comfortable?	It is Bulky	35 (23.8%)	105
	Difficulty in speech	25 (17%)	
	Difficulty in breathing	8 (5.4%)	
	Loose	25 (17%)	
	Causes nausea	12 (8.2%)	

It is worth noting that some of the participants did not respond to some of the items in the questionnaire. Almost one‐half of the patients (44.2%) reported having experienced trauma to teeth/mouth while actively involved in contact sports.

The majority of them were under fixed orthodontic treatment (76.9%) and the rest (23.1%) were under treatment with removable appliances. Of those, 34% had been wearing the appliance for more than 1 year, 42.2% for a time period of about 6 months to 1 year, and 23% had less than 6 months of duration of wear.

#### 3.2.1 Patients' perception and attitude toward MG during orthodontic treatment

**Figure 6:**
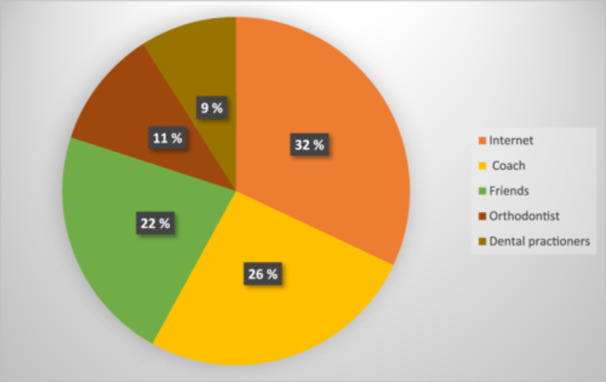
Sources through which orthodontic patients became acquainted with mouthguard.

More than two‐thirds of the included orthodontic patients (73%) reported that they had heard about MG before, primarily through internet sources as depicted in Figure 6.

The majority of orthodontic patients (82%) expressed their readiness to wear the MGs if recommended. However, more than one‐half (53%) showed reluctance to pay additional fees for the MGs.

**Figure 7:**
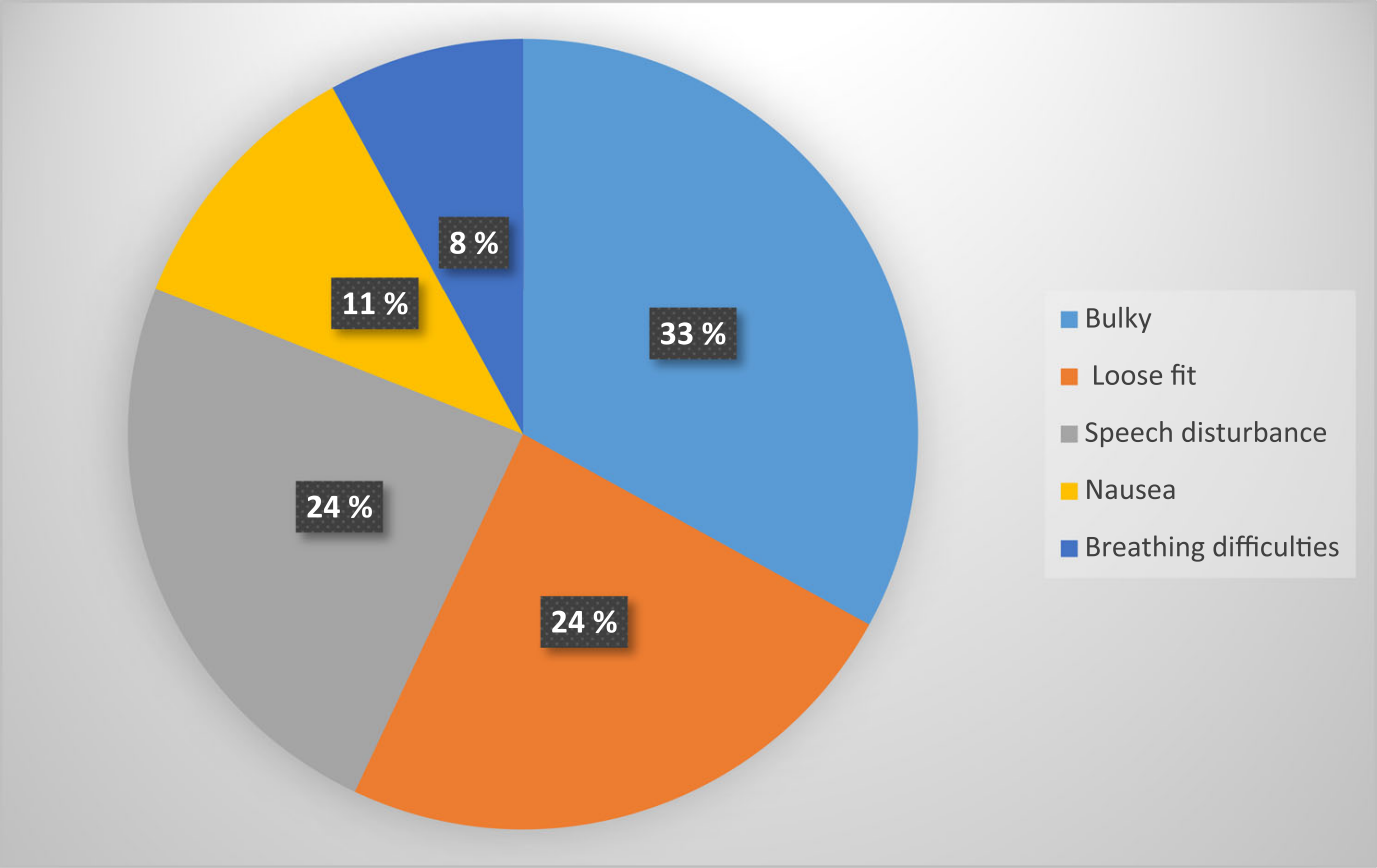
Factors contributing to discomfort due to mouthguard wear as perceived by orthodontic patients.

More than one‐third of participating orthodontic patients (39%) claimed that they routinely use MG while playing contact sports, and the majority of those (86%) believed that MG would offer protection from trauma resulting from contact sports. However, 89% of orthodontic patients perceived wearing MGs while involved in contact sports to be an uncomfortable experience for multiple reasons (Figure 7).

## 4 DISCUSSION

The risk of orofacial injuries in athletes involved in sports activities is inevitable. Undoubtedly, mouthguards offer protection to orofacial regions during sports activities. They provide a resilient and protective surface absorbing high‐impact energy from potentially traumatic blows, which otherwise would be transferred directly to the underlying dentition (Ferrari & De Medeiros, 2002). Evidence suggests poor acceptance of MGs among sports players with a lack of awareness regarding the risks of dental injury during sport (O'Malley et al., 2012; Tiryaki et al., 2017). Intriguingly, a recent survey conducted in the Middle East reported that 83% of the participants (parents of athletes) had no knowledge about the preventive aspect of MGs (Almalki et al., 2021).

The available evidence concerning the perception and acceptability of MGs among orthodontists and orthodontic patients is currently limited. Only a few studies have documented the perception of orthodontists in terms of the protective capabilities of MGs during contact sports (Bastian et al., 2020; Bussell & Barreto, 2014). To our knowledge, there is no quantitative study that explores and correlates both orthodontists and their own patients' standpoint on the use of MGs. Hence, this cross‐sectional study was conducted to report on the attitude, knowledge, and perception of orthodontists, dental practitioners, and orthodontic patients toward the use of MGs during contact sports activities.

In the present study, it was observed that around half of the orthodontic patients engaged in contact sports had experienced trauma. This appears to be higher than what has been reported for non‐orthodontic patients (less than 20%) (Meyfarth et al., 2023; Park et al., 2021). This discrepancy suggests that orthodontic patients may be more susceptible to traumatic injuries. The heightened risk of such injuries in orthodontic patients could be associated with pre‐existing malocclusions, particularly an increased overjet, or simply the presence of fixed orthodontic appliances, which increases the risk of oral soft‐tissue injuries when exposed to trauma due to sports due to the design of the appliance per se (Maeda et al., 2008).

In the current study, only 29% of dental clinicians enquired about their patients' involvement in contact sports as a part of history taking during their initial visit. This is in contrast to a recently conducted qualitative study, which reported that over half of the orthodontists or their staff intended to initiate discussion about contact sports involvement and mouthguards with patients, during the initial visits (Bastian et al., 2020; Bussell & Barreto, 2014).

In this investigation, it was noted that 46% of dental clinicians (45% orthodontists and 55% general dentists) recommended the use of MGs to their patients engaged in contact sports. Interestingly, fewer orthodontists recommended MGs to their patients compared to DP, which was in contrast with the findings of other surveys (Bastian et al., 2020; Bussell & Barreto, 2014) that reported more than 50% of orthodontists recommended MGs for their patients involved in contact sports. This difference in findings could potentially be explained by the relatively low number of dental clinicians who enquire about their patient's involvement in contact sports as part of their medical history during the initial visit. It is worth noting that patients are more likely to use MGs if their dental clinician educates them about the importance of mouthguard wear during contact sports activities (Bastian et al., 2020; Bussell & Barreto, 2014). Without asking about contact sports involvement, dental clinicians may overlook an important opportunity to educate patients on the importance of mouthguards.

The majority of dental clinicians displayed a positive attitude toward the use of MGs if their patients informed them about their involvement in contact sports during the course of treatment. However, it is worth noting that 64% of these clinicians would consider obtaining consent from patients or parents of the patients who are under‐aged before recommending MGs, primarily due to concerns about potential liabilities. This finding aligns with a previous study (Bastian et al., 2020) where some orthodontists believed it was necessary to inform their patients about the limitations of MGs in providing complete protection against traumatic injuries. According to dental clinicians, this approach is likely to ensure that patients have realistic expectations regarding the protective benefits of MGs and are well‐informed when making decisions about their use.

The majority of dental clinicians (79%) felt that the onus of recommending MGs to orthodontic patients is upon the orthodontists, among which less than half (45%) of orthodontists felt that it was their sole responsibility. This is consistent with a previous study, (Bastian et al., 2020) which found that most orthodontists believed that recommending MGs should be a shared responsibility of orthodontists, dentists, coaches, and parents rather than their sole responsibility. However, this standpoint among the participating dental clinicians did not align with that reported by the orthodontic patients. In the current investigation, patients reported that they were least likely to receive information about MGs from their dental clinicians. The fact that patients did not typically learn about MGs from their treating dental clinicians aligns with other studies (Bastian et al., 2020; Meyfarth et al., 2023), which found that coaches and clubs are the first to inform players under orthodontic treatment about MGs, rather than dental clinicians.

In contrast to other studies (Bastian et al., 2020; Bussell & Barreto, 2014), the current study found that stock MGs were the most preferred type of MG among dental clinicians. One plausible explanation for dental clinicians favoring stock‐type MGs could be their affordability, time factor, and widespread availability. However, it is important to note that stock‐type MGs lack retention features, relying on the athlete to maintain them in the mouth through biting, which does not guarantee an optimal fit, which in turn may explain the significant proportion of complaints about the utility of MGs reported by the patients. The majority of the orthodontic patients cited discomfort with the use of MGs owing primarily to its bulkiness followed by difficulty in articulation and fit.

Interestingly, few participating dental clinicians reported having an in‐office facility to fabricate custom‐made MGs. Their preference might be linked to the fact that custom‐made MGs offer better stability, retention, and user‐perceived comfort, which is a pivotal factor affecting compliance (Kalra et al., 2022). The potential financial advantages for clinicians could also play a role in their recommendation of custom‐made MGs. This is evident from a finding from the study, indicating that among the participating dental clinicians with an in‐house MG fabrication facility, the majority expressed an intention to charge their patients for custom‐made MGs. However, over half of the participating orthodontic patients were unwilling to bear additional expenses. This highlights that financial considerations may play a significant role in patients' preferences and adherence to using MGs. The potential cost associated with custom‐made MGs can be a barrier for some patients, which underscores the importance of considering affordability when discussing and recommending MGs to patients.

It is worth noting that a recent study involving orthodontic patients with fixed appliances participating in contact sports has found that custom‐made and mouth‐formed MGs offered a higher level of wearability in terms of patient preference compared to pre‐fabricated types (Kalra et al., 2022). Furthermore, custom‐made MGs have been proven better in absorbing impact and retaining their shape during laboratory impact testing when compared to pre‐fabricated or mouth‐formed types (Harrington et al., 2022). These findings suggest that there is a shifting paradigm concerning MG selection and that custom‐made MGs may offer superior performance and comfort.

The majority of the dental clinicians preferred their removable appliance patients to swap their appliances with MGs and fixed appliance patients to wear MGs while participating in contact sports during sports activities. This shows that dental clinicians believe in the protective ability of MGs regardless of the type of appliance, which was considered a barrier to recommendations by orthodontists in a previous study (Bussell & Barreto, 2014).

In the current study, some patients reported experiencing trauma even though they were wearing mouthguards. This could be attributed to several possible reasons such as inadequate fit and patient discomfort (as reported by some of the participating orthodontic patients), quality of MGs, improper use, impact severity, and wear and tear.

One strength of the current study is the inclusion of both clinicians (caregivers) and their patients (recipients of care) allowing the exploration of both clinicians' attitudes and patients' perceptions to provide a more holistic view of the situation. Furthermore, this study did not restrict patients' participation in a specific sport type.

Limitations of this study include utilizing a convenience sampling approach to select the participants, which may affect the generalizability of the study results. Also, the survey did not consider the potential impact of socioeconomic status on the affordability of MGs. Additionally, included patients were treated with different appliance types with variable severity of malocclusion, which may influence patients' experiences and outcomes.

## 5 CONCLUSION

- Most of the dental clinicians expressed a positive attitude and belief toward the protective ability of MGs.
- The majority of dental clinicians did not enquire but relied on patients to proactively disclose their sports activities before recommending mouthguards.
- Patients primarily acquired knowledge about mouthguards from internet sources, rather than from their dental clinicians.
- Patients expressed a willingness to wear mouthguards if they were recommended by dental clinicians, whom they regarded as trusted professionals.
- Stock‐type MGs were the most preferred among the participating clinicians, reflected by the perceived discomfort of the orthodontic patients.
- Patients expressed their reluctance to incur additional costs for MGs.

## 6 CLINICAL IMPLICATION

Given the increasing incidence of traumatic injuries, it has become imperative to implement regulations and strategies to make the use of MGs mandatory, especially in contact sports. Dental clinicians, particularly orthodontists, play a significant role in developing strategies to promote the adoption of MGs among athletes undergoing orthodontic treatment. Consideration of the financial status of the patients is essential to enhance adherence to MGs.

## AUTHOR CONTRIBUTIONS

**Shailaja Raghavan**: Methodology; data analysis; writing—original draft. **Elham S. Abu Alhaija**: Conceptualization; methodology; data analysis; writing—original draft. **Yousef Nasrawi**: Methodology; data collection. **Susan Al‐ Khateeb**: Methodology; data collection. **Samer Sunna**: Methodology; data collection. All the authors contributed to revision and approval of the final manuscript.

## CONFLICT OF INTEREST STATEMENT

The authors declare no conflict of interest.

## ETHICS APPROVAL AND CONSENT TO PARTICIPATE

The protocol was approved by the Deanship of research/Jordan University of Science and Technology (Research ID20070048).

## Data Availability

The data that support the findings of this study are available from the corresponding author upon reasonable request.
